# Discrimination of geographical origin of cultivated *Polygala tenuifolia* based on multi-element fingerprinting by inductively coupled plasma mass spectrometry

**DOI:** 10.1038/s41598-017-12933-z

**Published:** 2017-10-03

**Authors:** Yunsheng Zhao, Xiaofang Ma, Lingling Fan, Fuying Mao, Hongling Tian, Rui Xu, Zhe Cao, Xinhui Zhang, Xueyan Fu, Hong Sui

**Affiliations:** 1 Ningxia Medical University Pharmacy College, Yinchuan, 750004 Ningxia China; 2 Ningxia Research Center of Modern Hui Medicine Engineering and Technology, Yinchuan, 750004 Ningxia China; 3 Ministry of Education Key Laboratory of Modern Hui Chinese Medicine, Yinchuan, 750004 Ningxia China; 4 Institute of Industrial Crop Research, Shanxi Academy of Agricultural Sciences, Fenyang, 032200 Shanxi China

## Abstract

Inorganic elements are important components of medicinal herbs, and provide valuable experimental evidence for the quality evaluation and control of traditional Chinese medicine (TCM). In this study, to investigate the relationship between the inorganic elemental fingerprint and geographical origin identification of cultivated *Polygala tenuifolia*, 41 elemental fingerprints of *P*. *tenuifolia* from four major polygala-producing regions (Shanxi, Hebei, Henan, and Shaanxi) were evaluated to determine the importance of inorganic elements to cultivated *P*. *tenuifolia*. A total of 15 elemental (B, Ca, Cl, Cu, Fe, K, Mg, Mn, Na, N, Mo, S, Sr, P, and Zn) concentrations of cultivated *P*. *tenuifolia* were measured using inductively coupled plasma mass spectroscopy (ICP-MS). The element composition samples were classified by radar plot, elemental fingerprint, and multivariate data analyses, such as hierarchical cluster analysis (HCA), principle component analysis (PCA), and discriminant analysis (DA). This study shows that radar plots and multivariate data analysis can satisfactorily distinguish the geographical origin of cultivated *P*. *tenuifolia*. Furthermore, PCA results revealed that N, Cu, K, Mo, Sr, Ca, and Zn are the characteristic elements of cultivated *P*. *tenuifolia*. Therefore, multi-element fingerprinting coupled with multivariate statistical techniques can be considered an effective tool to discriminate geographical origin of cultivated *P*. *tenuifolia*.

## Introduction

*Polygala tenuifolia* (family: Polygalaceae) is a perennial herb that has been cultivated throughout East Asia (*P*. *tenuifolia* in Korea, Wonji in Korean, Onji in Japanese, and Yuanzhi in Chinese)^1^. This herb is also distributed in the different regions in China, such as Shanxi, Shaanxi, Hebei, Henan, Gansu, Qinghai, Heilongjiang, Liaoning, Inner Mongolia, Jiangxi, Jiangsu, and Sichuan^2^. *P*. *tenuifolia* is a well-known TCM prescribed for amnesia, neurasthenia, palpitation, asthma, rhinitis, and insomnia^3^. Modern pharmacological studies have demonstrated that Polygala has a wide range of biological activities, such as improved learning and memory, anti-aging, antimicrobial, expectorant, and increased uterine contractions and muscle tension. *P*. *tenuifolia* primarily contains oligosaccharides^4,5^, xanthones^6^, and saponins^7^. Moreover, the saponins from *P*. *tenuifolia* have a complex chemical structure^8^. Xanthones have displayed antitumor^9^, antimicrobial^10^, anti-thrombotic^11^, and anti-inflammation activities. As a widely used medicinal plant, *P*. *tenuifolia* has attracted attention due to its superior pharmaceutical properties^12^.

With growing consumption and sales of *P*. *tenuifolia* in recent years, the wild resource has been worsened by overexploitation^13^, leading to the decline of wild resources for TCM. Alternatively, the cultivated *P*. *tenuifolia* is used as a medicine, but it has a low yield and is time consuming because it needs to be harvested by dredging after being planted for a few years. Poor quality cultivated *P*. *tenuifolia* is increasingly becoming available in the market, because the cultivated *P*. *tenuifolia* comes from non-authentic producing regions. Therefore, geographical origin, which affects the quality and efficacy, and authenticity of cultivated *P*. *tenuifolia* are highly important. Identifying the geographical origin of TCM is crucial because it determines quality^14^. The differences between authentic and non-authentic TCMs are invisible to the naked eye; therefore, systematic control of the authenticity TCM relies on chemical analysis^15^. The current elemental fingerprint study differentiated the crops and focused on the organic composition^15^; however, only a few fingerprints were reported on the inorganic elements or differentiation of TCMs. Many investigations revealed that the contents of inorganic elements in TCM play a significant role in the biological activity and greatly affects quality^16^. Therefore, constructing inorganic elemental fingerprints is valuable in identifying the geographical origin of TCM^17^. Elemental fingerprint techniques have been used in many plant studies based on element composition and multivariate statistical analysis. ICP-MS, as an element-specific detector, has several advantages, such as wide linear range, high sensitivity, multi-elements, and multi-isotopes detection ability^18^. These factors help determine various elements for qualitative, quantitative, and semi-quantitative analyses. The analytical approach used in this study is based on ICP-MS due to its aforementioned advantages.

This study did not focus on the identification of cultivated *P*. *tenuifolia* using the elemental composition profiles. Instead, it determined the 15 inorganic elements in cultivated *P*. *tenuifolia* using ICP-MS and investigated the elemental compositions. This study discriminated cultivated *P*. *tenuifolia* from different regions combined with multivariate analysis, and further established a reliable method for differentiating cultivated *P*. *tenuifolia*. The corresponding results demonstrated that the combination of inorganic elemental fingerprint with multivariate statistical analysis is a promising approach to discriminate the geographical origin of cultivated *P*. *tenuifolia*.

## Results

### Element concentrations of cultivated *P*. *tenuifolia* samples

A total of 41 cultivated *P*. *tenuifolia* samples were collected from four major polygala-producing four provinces (Shaanxi, Hebei, Henan, and Shanxi) in China (Supplementary Table S1 and Fig. S1). To investigate the relationship between the inorganic elemental fingerprints and the geographical origin identification of cultivated *P*. *tenuifolia*, 15 inorganic elements were determined in cultivated *P*. *tenuifolia* samples by ICP-MS^15^. Moreover, the elemental contents of cultivated *P*. *tenuifolia* are listed in Supplementary Table S2.

The mean concentration of elements in cultivated *P*. *tenuifolia* is classified as follows: Mo < Cu < B < Zn < Mn < Sr < Na < Cl < Fe < S < Mg < P < K < N < Ca. Furthermore, the general order of the concentrations is Cu, Zn, Mn, Fe, Mg, and Ca, which corresponds to those of a previous study^19^. This sequence is associated with the pharmacodynamic material basis of cultivated *P*. *tenuifolia*.

### Establishment and evaluation of the elemental fingerprint of *P*. *tenuifolia* grown in different locations

**Figure 1:**
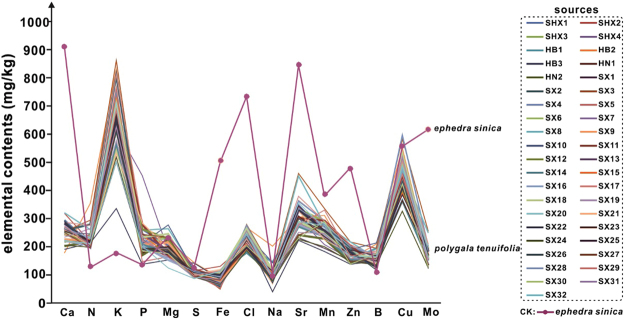
The elemental contents of 41 cultivated *P*. *tenuifolia* samples and *ephedra sinica*.

To visually demonstrate the distribution rules of the elemental contents, according to the results of the ICP-MS, the content distribution curves of 15 elements from 41 samples were drawn together in one chart (Fig. 1), as *ephedra sinica* sample^20^. For drawing convenience, some elements were expanded or narrowed to the same order of magnitude (B, Zn, Mn, and Sr expanded tenfold; Mo and Cu expanded 100-fold; K, P, Mg, S, and Fe reduced tenfold; Ca and N reduced 100-fold). The contents of individual elements, such as Fe, Cl, Zn, and Mo, from cultivated *P*. *tenuifolia* samples were maintained at a certain range, but different from that from *Ephedra sinica* sample. Other elemental content, such as Ca, K, Mg, Na, Sr, Mn, B, and Cu, displayed a similar peak shape in the chart. Obviously, the content of the individual elements, such as Ca, Fe, Cl, Sr, Zn, and Mo, from the cultivated *P*. *tenuifolia* samples differed greatly from that of the *ephedra sinica* sample, in which Ca content had expanded more than 3.6 fold from the average 250 mg/kg of cultivated *P*. *tenuifolia* samples to 900 mg/kg of *ephedra sinica* sample.

Studies showed that the differences of elemental contents are associated with diverse origins, the elements in cultivated *P*. *tenuifolia* samples maintained a certain balance compared with the peak of *Ephedra sinica*, which presented a completely different shape. Therefore, a commonality of elemental fingerprint exists, which determines the difference between *P*. *tenuifolia* and other medicinal plants. This discovery promotes the application of inorganic elemental fingerprints for determining the geographical origin of cultivated *P*. *tenuifolia*. This study suggested that the elemental fingerprint could be used to identify the authenticity of cultivated *P*. *tenuifolia*.

### Distribution of the cultivated *P*. *tenuifolia* samples according to HCA

**Figure 2:**
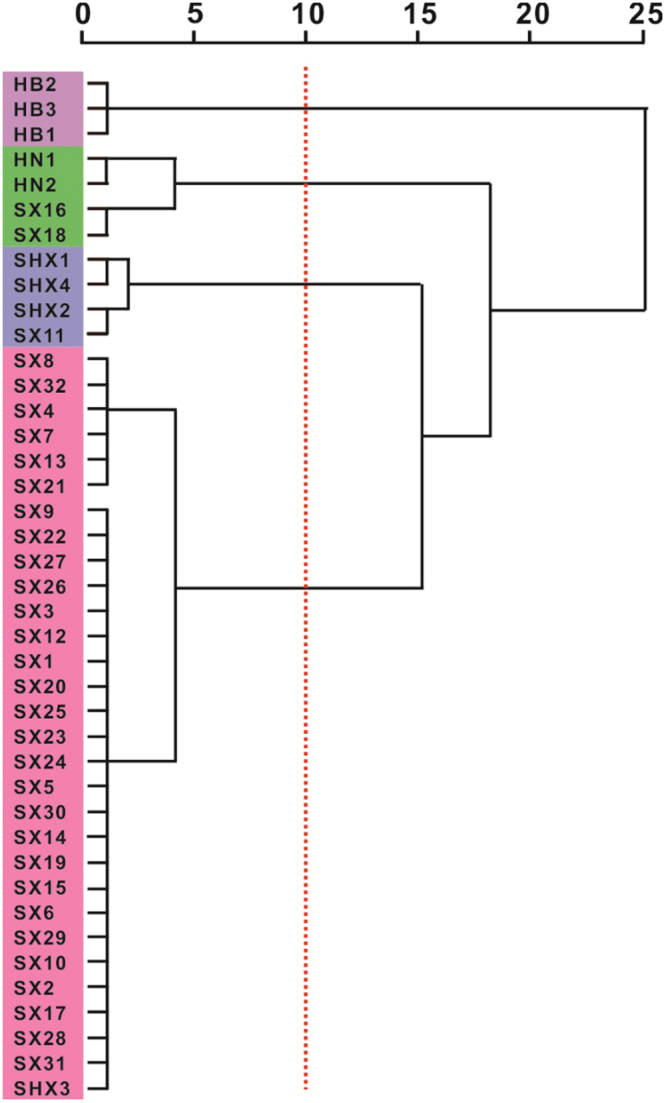
Dendrograms of hierarchical cluster analysis (HCA) for 41 cultivated *P*. *tenuifolia* samples from different major polygala-producing origin.

To improve the visualization of the relative distribution of the cultivated *P*. *tenuifolia* samples according to their geographical origins, HCA was performed using the first three discriminant normalization scores^21^. Cultivated *P*. *tenuifolia* samples from different regions were separated into four clusters based on the dendrograms cut at a distance of 3 (Fig. 2). The first cluster was composed of samples from the Hebei province, including Anguo, Neiqiu, and Xinglong, and the second cluster mainly was comprised of cultivated *P*. *tenuifolia* samples (Xingyang and Mengjin) from Henan, and two cultivated *P*. *tenuifolia* samples (Wanan and Pinglu) from Shanxi. The third cluster included Suide, Pucheng, and Zizhou from Shaanxi province, and Yicheng from Shanxi province. The fourth cluster was composed of 30 cultivated *P*. *tenuifolia* samples. A total of 29 cultivated *P*. *tenuifolia* samples were collected from the Shanxi province and only one cultivated *P*. *tenuifolia* sample from Shaanxi. The cluster analysis revealed that Hebei-cultivated *P*. *tenuifolia* samples are found in other regions. Some of the cultivated *P*. *tenuifolia* samples from different regions were clustered together^22^; notably, samples from Henan and Shanxi have similar elemental contents. For example, both of Shanxi- and Hebei-cultivated *P*. *tenuifolia* samples have significantly high K content. Overall, the cluster results are generally in agreement with the actual origin of cultivated *P*. *tenuifolia* samples. Results implied that elemental information is suitable for classifying the cultivated *P*. *tenuifolia* samples from different regions. However, due to the geographical origin of the cultivated *P*. *tenuifolia* samples, an overlap between Shaanxi and Shanxi occurs. Therefore, the radar plot analysis was used to further study the identification of the cultivated *P*. *tenuifolia* from different regions.

### Distinguishing geographical origin of cultivated *P*. *tenuifolia* samples by radar plot

**Figure 3:**
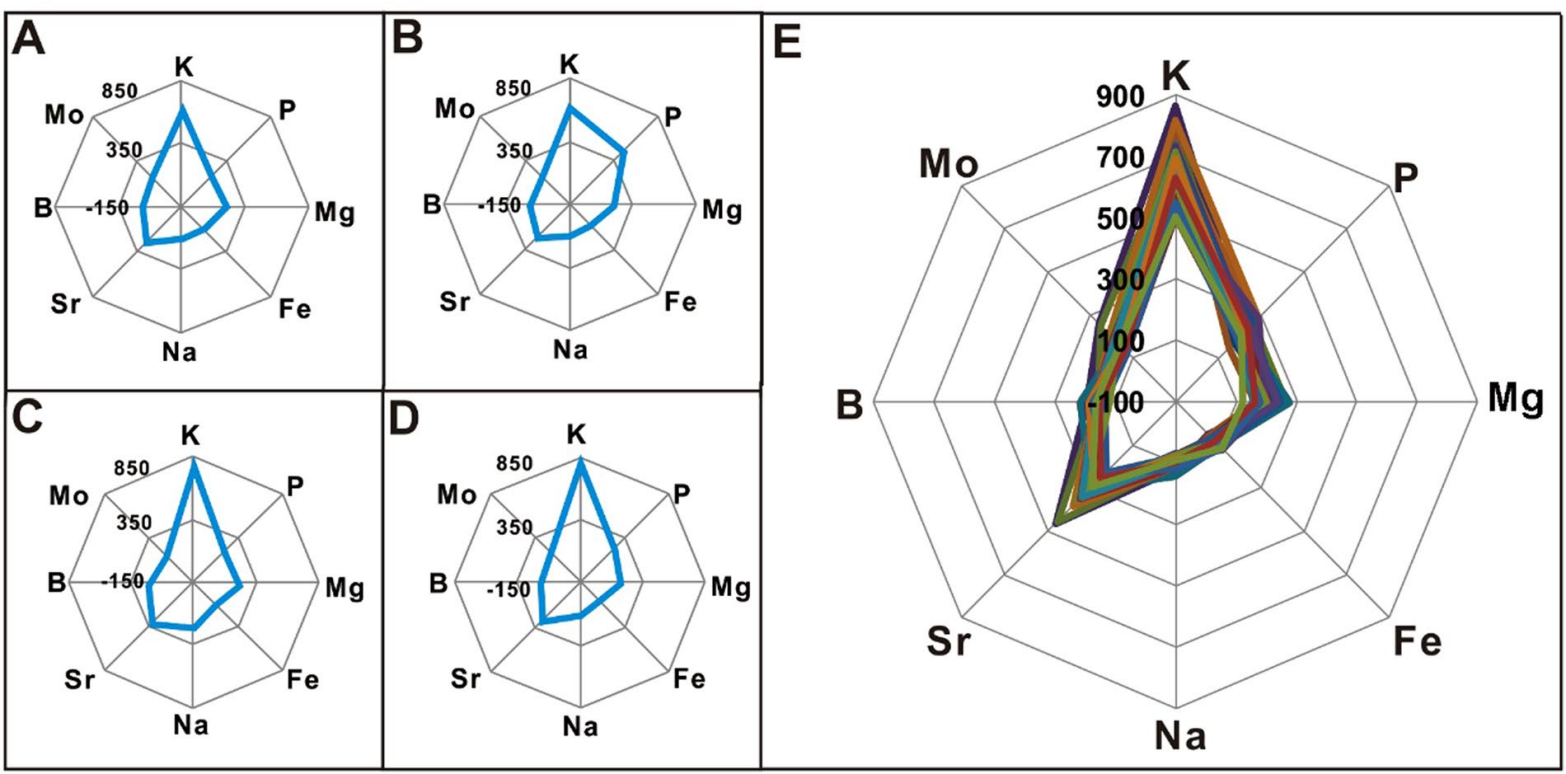
Radar plots showing the difference of geographical origins in term of mean concentrations of elements (K, P, Mg, Fe, Na, Sr, B, and Mo) in various cultivated *P*. *tenuifolia* samples (**A**. Shanxi, **B**. Henan, **C**. Shaanxi and **D**. Hebei province) and geographical origin in term of relative concentrations of elements (K, P, Mg, Fe, Na, Sr, B, and Mo) in 32 cultivated *P*. *tenuifolia* samples from different regions of Shanxi province (**E**).

A radar plot based on the elemental composition has been used for distinguishing the geographical origin of cultivated *P*. *tenuifolia* samples. This method allows the use of a simple routine and rapid discrimination. For ease of comparison, a radar plot was performed to preliminarily classify cultivated *P*. *tenuifolia* origins based on the mean concentrations of the eight elements (K, P, Mg, Fe, Na, Sr, B, and Mo). These concentrations showed high relative standard deviation (RSD) in each cultivated *P*. *tenuifolia* sample. The distributions of the elemental patterns of cultivated *P*. *tenuifolia* samples from various provinces showed different characteristic patterns, as shown in Fig. 3. This study showed that some elements in cultivated *P*. *tenuifolia* vary in different regions^23^. Therefore, this method distinguishes the geographical origin of different cultivated *P*. *tenuifolia* samples. The cultivated *P*. *tenuifolia* samples are found in the two nearby provinces, namely, Shanxi and Hebei. This species has a significantly higher K content and is easily distinguished compared with the cultivated *P*. *tenuifolia* from the other two regions. This finding corresponded to the result of HCA (Shanxi and Hebei gather in clusters). Radar plots analysis was performed for 32 cultivated *P*. *tenuifolia* samples collected from different regions of the Shanxi province. Radar plots of 32 cultivated *P*. *tenuifolia* samples from different regions of Shanxi province illustrated that the distribution of elemental patterns shows the same characteristics. Moreover, the pattern is the same for all the cultivated *P*. *tenuifolia* samples from the same province (Fig. 3E). This finding indicated that the cultivated *P*. *tenuifolia* samples from the same province have a similar growth environment, including light, temperature, and humidity^24–26^. However, visual determination was the only result obtained by the radar plot analysis and the lack of definite index describing the exact differences decreased the credibility of the results. Therefore, the principal component analysis (PCA) was utilized in the following studies for actual discrimination.

### Analysis of elements influencing the discrimination of *P*. *tenuifolia* by Principle component analysis

**Figure 4:**
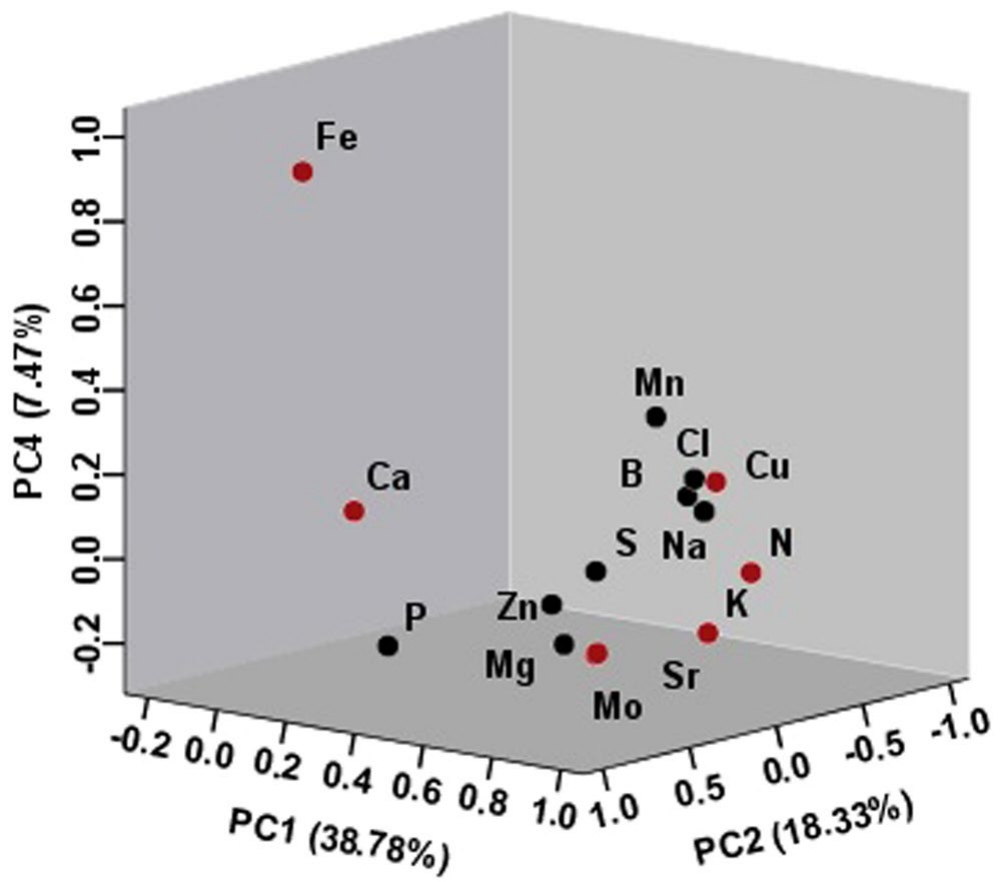
A three-dimensional PCA plot (PC1, PC2, and PC4) generated from principal component analysis (PCA) for the concentration of 15 elements. The red dots indicate the characteristic elements (N, K, Mo, Sr, Cu, Ca, Fe) of the cultivated *P*. *tenuifolia* as shown in the PCA plot.

Principle component analysis (PCA) was accomplished by factor analysis in SPSS software^27^. PCA was performed on elemental concentrations according to 15 variables to classify all cultivated *P*. *tenuifolia* samples from different geographical origins. The results demonstrated that the first four principal components (PC1-4), with eigenvalues >1 had 74.024% of the total variability among the 15 variables in the original data, where in PC1, PC2, PC3, and PC4 contributed 38.78%, 18.33%, 9.45%, and 7.47% of the total variance, respectively (Table 1). This finding showed that a four-factor model could explain 74.024% of the test data. The first three principal component loading plot (Fig. 4) displayed that the N, Cu, K, Mo, Mn, and Sr contents have the highest weights in PC1; Ca content loaded highly in PC2; all element contents have a low PC3; Fe was the dominating feature content in PC4. The contribution of more than 74.024% varied from PC1 to PC4; therefore, the elements (N, Cu, K, Mo, Mn, Sr, Ca, and Fe) were regarded as the characteristics of inorganic elements in the cultivated *P*. *tenuifolia*. These elements may be identified as the most powerful indicators of cultivated *P*. *tenuifolia*. Furthermore, the trace elements Fe, Mn, and Cu are indicators of the geographical origin of plant samples due to their different concentrations in soils and effective uptake by plants^28^.

**Table 1:** The vectors and cumulative contribution of variance of the first four principal components.

Items	Principal component			
	1	2	3	4
Ca	0.253	0.820	0.207	0.158
N	0.784	−0.416	0.001	−0.038
K	0.849	−0.038	0.247	−0.134
P	0.098	0.317	0.553	−0.234
Mg	0.415	−0.070	0.261	−0.226
S	0.497	−0.092	−0.471	−0.043
Fe	−0.171	0.275	0.185	0.846
Cl	0.672	−0.312	−0.432	0.178
Na	0.580	−0.550	0.457	0.065
Sr	0.801	0.506	−0.072	−0.132
Mn	0.733	0.030	0.170	0.369
Zn	0.720	0.607	−0.129	−0.019
B	0.506	−0.600	0.371	0.085
Cu	0.762	−0.258	−0.305	0.19
Mo	0.797	0.510	−0.081	−0.137
Variance (%)	38.782	18.330	9.446	7.466
Cumulative variance (%)	38.782	57.113	66.558	74.024

The PCA score plot (Supplementary Fig. S2A) illustrates a separation pattern of cultivated *P*. *tenuifolia* samples, whereas the corresponding loading plot (Supplementary Fig. S2B) describes the variables related to the separation. Elements, such as Sr, Mo, K, N, Cu, Mn, and Zn, controlled the discrimination of cultivated *P*. *tenuifolia* samples. Only 38.78% and 18.33% of the dataset were explained by the principle components 1 and 2, respectively.

To improve the discrimination, PCA was applied on the concentration values of 15 elements to classify the cultivated *P*. *tenuifolia* samples from Shanxi province. As shown in the results of the score and the loading plots in Supplementary Fig. S2C,D, the cultivated *P*. *tenuifolia* samples from the southern regions are easily distinguished. Elements, such as N, K, S, Sr, and Mo, influenced the separation of cultivated *P*. *tenuifolia* samples. The plots were defined by the principal components 1 and 2 and explained by the 45.01% and 15.12% variance. The results obtained by PCA did not allow for good discrimination of the geographical origin of cultivated *P*. *tenuifolia*. Therefore, DA could be applied for improved separation.

### Discrimination of geographical origin of cultivated *P*. *tenuifolia* based on Discriminant analysis (DA)

DA of elemental concentrations measured by ICP-MS in the cultivated *P*. *tenuifolia* samples from different geographical origins were applied to classify groups; then, the discriminant functions correlated with the variables were obtained. The coefficients and cumulative contribution of the different variables in the discriminant functions for cultivated *P*. *tenuifolia* classification are shown in Table 2. In this study, the calculation was performed using seven variables (Ca, P, Mg, Fe, Na, Mn, and B) to classify all cultivated *P*. *tenuifolia* samples.

**Table 2:** Coefficient and cumulative contribution of the discriminant functions.

Elements	For all cultivated *P*. *tenuifolia* (7 variables)			Elements	For Shanxi cultivated *P*. *tenuifolia* (6 variables)	
	F1	F2	F3		F1	F2
Ca	0.401	0.752	0.864	Ca	−0.075	0.359
Fe	−0.209	−0.718	−0.173	Fe	0.38	0.29
Mg	−0.266	−0.551	−0.069	Mg	−0.459	−0.303
Mn	−0.064	0.615	0.265	Mn	0.775	−0.232
B	0.053	−0.278	0.843	B	−0.774	−0.012
Na	0.858	0.638	−0.962	Zn	−0.696	0.119
P	−0.203	0.802	−0.503			
variance %	78.8	15.5	5.7	variance %	68.2	31.8
Cumulative variance %	78.8	94.3	100	Cumulative variance %	68.2	100

**Figure 5:**
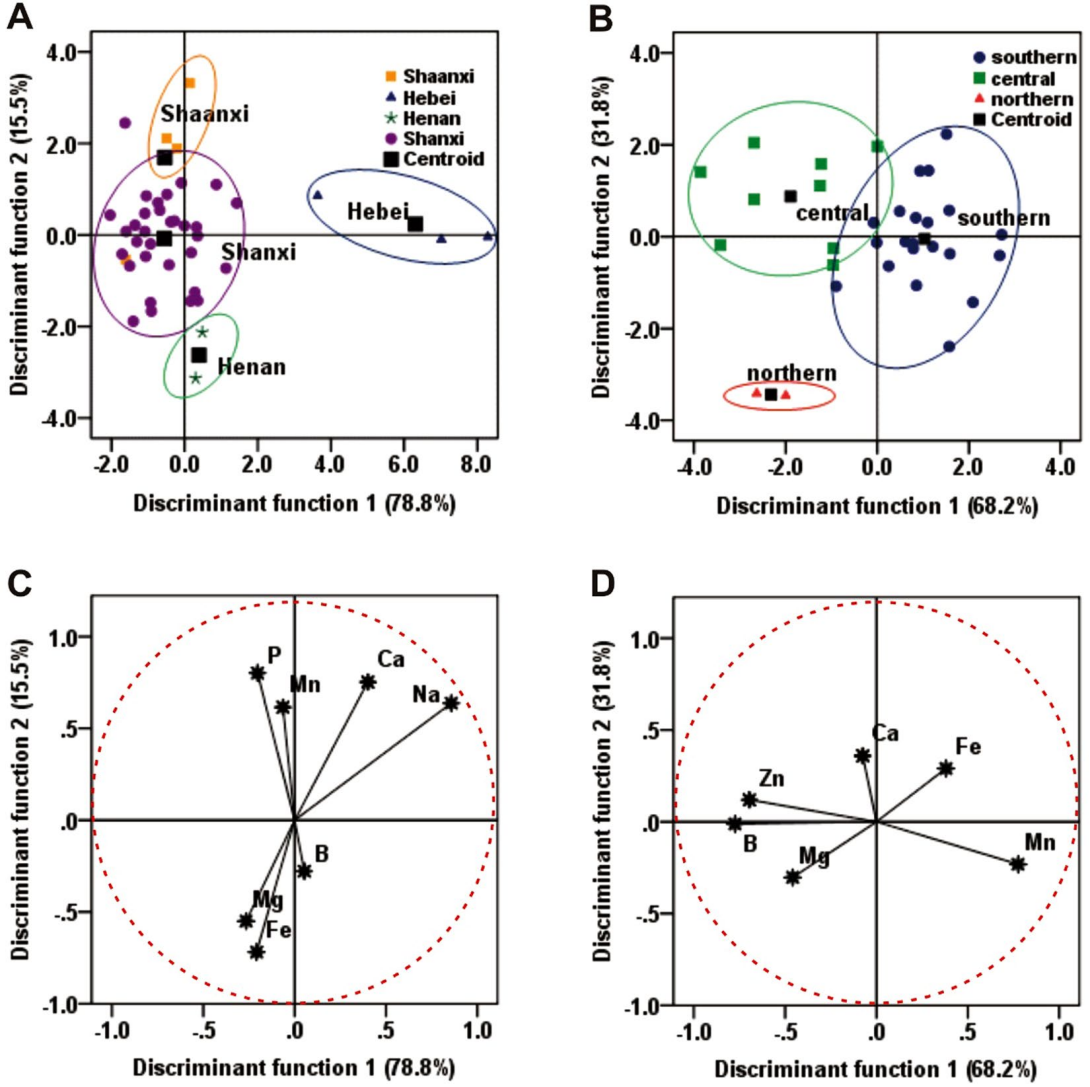
Discrimination analysis for 41 cultivated *P*. *tenuifolia* samples from different major polygala-producing origin. (**A**) Scatter diagram of cultivated *P*. *tenuifolia* samples from four different provinces (Shaanxi, Hebei, Henan, and Shanxi); (**B**) Scatter diagram of cultivated *P*. *tenuifolia* samples from Shanxi province (southern, northern, and central regions). analyzed according to different regions obtained through the two discriminant functions after discriminant analysis. (**C**) Correlation chart between the selected variables and the discriminant functions for all cultivated *P*. *tenuifolia* samples; (**D**) Correlation chart between the selected variables and the discriminant functions for Shanxi cultivated *P*. *tenuifolia* samples.

Distribution patterns of all cultivated *P*. *tenuifolia* samples were performed according to their origins in the plot defined by the discriminant functions (Fig. 5A). The variations between groups were explained by the discriminant functions 1 (78.8%) and 2 (15.5%). The cultivated *P*. *tenuifolia* from Shanxi, Henan, Shaanxi, and Hebei were completely separated.

DA was further performed on the elemental concentrations based on the six variables (Mg, Fe, Mn, Zn, B, and Mo) of the cultivated *P*. *tenuifolia* samples (Table 2) found in the different regions of Shanxi, as shown in Fig. 5B. The distribution pattern of Shanxi-cultivated *P*. *tenuifolia* defined by the discriminant functions 1 and 2 was plotted and was explained by the 68.2% and 38.1%, respectively. The plot shows a clear classification of cultivated *P*. *tenuifolia* origins from the three regions of Shanxi (southern, northern, and central).

Figure 5C represents the correlation chart of loadings for the selected elements, in the plane designed by the first two discriminant functions (F1 and F2). F1 expressed 78.8% of the variance, which provided the main separation between all cultivated *P*. *tenuifolia* regions and has a strong positive correlation with Ca, Na, and B; whereas F2 (15.5% of the variance) has a positive correlation with Mn and P concentrations. The correlation between Fig. 5A,C (Ca, B, P, and Na**)** was observed as the most useful variable for discriminating all cultivated *P*. *tenuifolia* samples from four provinces. This study showed that the cultivated *P*. *tenuifolia* samples from four different provinces were plotted in various spaces.

By applying DA to cultivated *P*. *tenuifolia* samples, the researchers were able to distinguish the southern, northern, and central regions of Shanxi province and notice that their projections fall in different dimensions of the chart. The main separators between these three regions were the elements Ca, Fe, and Mn (Fig. 5D). F1 expressed 68.2% of the variance, which provide the main separation between Shanxi regions and has a strong positive correlation with Fe and Mn, whereas F2 (31.8% of the variance) has a strong correlation with Ca, Fe, and Zn concentrations.

In this study, the multivariate statistic approach was used to verify the correlation between the compositions of cultivated *P*. *tenuifolia* inorganic elements and the authentic regions. The P, Mn, Ca, Na, Mg, Fe, and B were used to classify all cultivated *P*. *tenuifolia* samples according to their geographical origins. Moreover, Mn, Ca, Mg, Fe, Zn, and B were applied to recognize the different regions of the Shanxi province. Elements were classified using the multivariate statistic approach, thereby confirming the relationship between cultivated *P*. *tenuifolia* authentic regions and inorganic elements.

To check the reliability of the developed classification model, cross-validation method was operated to compute the classification and probability of cultivated *P*. *tenuifolia* samples^29^. Table 3 summarizes the observation of the cross-validation results together with the classification of cultivated *P*. *tenuifolia* samples using the DA model. The results showed that 80.5% of the cultivated *P*. *tenuifolia* samples (Shanxi, Henan, Shaanxi, and Hebei) and 100% of the 32 Shanxi cultivated *P*. *tenuifolia* samples (southern, northern and central regions) were correctly classified. These results were similar to findings of the previous studies; therefore, the multi-element analysis is a reliable fingerprinting analytical strategy for authenticating cultivated *P*. *tenuifolia*.

**Table 3:** Classification Results of cultivated *P*. *tenuifolia* samples using discriminant analysis.

Origin	Assigned origin for all cultivated *P*. *tenuifolia* samples with 7 variables					
	Shaanxi	Hebei	Henan	Shanxi	Total	Correct (%)
Shaanxi	4	0	0	0	4	100.0
Hebei	0	3	0	0	4	100.0
Henan	0	0	2	0	2	100.0
Shanxi	2	0	6	24	32	75.0
Total	6	3	8	24	41	80.5
**Origin**	**Assigned origin for 32 cultivated** ***P***. ***tenuifolia*** **samples from shanxi with 6 variables**					
	**Southern**	**Central**	**Northern**	**Total**	**Correct (%)**	
Southern	21	0	0	21	100.0	
Central	0	9	0	9	100.0	
Northern	0	0	2	2	100.0	
Total	21	9	2	32	100.0	

## Discussion

### Variation in element contents and the bioactivity of *P*. *tenuifolia*

The 15 elemental fingerprints of *P*. *tenuifolia* are consisted of macro elements (N, K, and P), secondary elements (Ca, Mg, and S), and trace elements (Fe, Mn, B, Zn, Cu, Mo, Cl, and Sr). A recent study reported that Chinese herbal medicines contain trace elements that are essential to the human body. Moreover, trace elements in other TCM and their active ingredients have different degrees of relationship^30,31^. Based on the results of the multi-element analysis, the absorption of trace elements in TCM is affected by their chemical compositions^32,33^. Moreover, these elements exhibit complex interactions^34^. This study showed that the contents of Ca and N are high in 41 cultivated *P*. *tenuifolia* samples, whereas the RSD of the two elements is low. This result indicated that the contents of these two elements have a narrow range of variation and thus are relatively stable. Ca is required for plant cell division and enlargement, and its insufficiency can generate leaves deformity, terminal buds death, and reduce root growth. Ca is also important for human healthy bones and teeth, muscle contraction, nerve impulse stimulation, and ion transport. N is the component of amino acids, nucleic acids, and vitamins in the plants or animals. The deficiency of N can generate human malnutrition, inactivity and failure to grow^35^. Quantitative Ca and N may be significant for the yield and biological activity of cultivated *P*. *tenuifolia*.

### Elements and geographical origins of TCM

Genuineness of TCM is related to the climate and environment and the geological environment, especially the soil geochemical characteristics^36^. Environmental biogeochemical and trace elements provide scientific guidance for the correct introduction and transplantation of TCM^36^. The elemental fingerprints of *P*. *tenuifolia* from different provinces (Fig. 1) displayed a similar peak shape compared with *E*. *sinica*, thereby indicating the element in cultivated *P*. *tenuifolia* maintained a commonality, which could identify the difference between *P*. *tenuifolia* and other medicinal plants. At the same time, The element fingerprint peak height had a wide range, indicating the cultivated *P*. *tenuifolia* in different production regions have a great difference. In this study, the combination of multi-element fingerprinting and multivariate statistical techniques can be used as an effective tool for distinguishing the geographical origins of cultivated *P*. *tenuifolia*. So the elemental fingerprint could be used to identify the authenticity of cultivated *P*. *tenuifolia*.

## Materials and Methods

### Materials

In this study, the roots of cultivated *P*. *tenuifolia* were collected in August and October2013 (Supplementary Table S1). A total of 41 cultivated *P*. *tenuifolia* samples were gathered from the four provinces (Shaanxi, Hebei, Henan, and Shanxi) in China, as shown in Supplementary Fig. S1. Professor Yunsheng Zhao and assistant research fellow Hongling Tian identified the plants as the roots of authentic *P*. *tenuifolia*.

The ICP-MS element standard stock solutions for mineral elements were provided by Beijing General Research Institute for Nonferrous Metals in China. All glassware and plastic ware were cleaned with nitric acid and rinsed with deionized water prior to the procedure.

### Methods: ICP-MS Measurements

The concentrations of essential elements in plant samples were determined by inductively coupled plasma mass spectrometry (NexION 300D, PerkinElmer Instrument Co., U.S.) following the modified JIS K0133-2007 Method (Japanese Industrial Standards Committee 2007). The instrument parameters were optimized as follows: plasma gas flow rate of 18.0 L/min, carrier gas flow rate of 1 L/min, auxiliary flow rate of 1.20 L/min, dwell time of 50.0 ms, radio frequency power of 1600 W, sample uptake of 1.0 ml/min, scan time of 20 s, and integral time of 1 s.

### Statistical Analysis

In this study, the analytical data were manipulated using the Excel 2010 spreadsheet. The SPSS 21.0 software was used for pattern recognition computations. Pattern recognition methods were applied involving the multivariate data analysis. The discrimination and authenticity of the cultivated P. *tenuifolia* samples were carried out by the following multivariate data analysis (chemometric) techniques: hierarchical cluster analysis (HCA), principle component analysis (PCA), and discriminant analysis (DA). The statistical analysis used in this study is as follows:

#### The establishment of element fingerprint and radar plot analysis

The elemental fingerprints and radar plots were analyzed. The statistical analysis was performed using Microsoft Office Excel 2010.

#### Hierarchical cluster analysis (HCA)

HCA is an unsupervised classification procedure that involves measuring the similarity between samples to be clustered. Hierarchical cluster analysis (HCA) of samples was performed using the selected chemical descriptors as variables, the Ward’s method as the amalgamation rule, and the squared Euclidean distance as the similarity measurement. Samples were grouped in clusters based on their nearness and similarities^37^. The groups were represented by the branches of the dendrogram. The dendrogram showed the different groups at a normalized or rescaled distance. The between-group linkage method was the clustering method used in this study.

#### Principal components analysis (PCA)

PCA is a projection method that allows for easy visualization of all information contained in the data set. PCA reduces the dimensionality of the data matrix and transforms original variables into principal components (PCs)^38^. By plotting the PCs, one can view the interrelationships between different samples and examine the grouping of samples. Finally, PCAs quantify the amount of useful information, as opposed to meaningless variations, contained in the data^39^.

### Discriminant analysis (DA)

DA is a widely known supervised pattern recognition method for classifying the maximum variance between groups and minimum variance within the groups by creating new variables, which are linear combinations of the original variables^40^. This classification procedure maximizes the variance between categories and minimizes the variance within categories.

### Data Availability

All data generated or analysed during this study are included in this published article (and its Supplementary Information files).

## Electronic supplementary material

Supplementary Information
