# Supplementary material for: Discrimination of geographical origin of cultivated Polygala tenuifolia based on multi-element fingerprinting by inductively coupled plasma mass spectrometry
Source: Sci Rep. 2017 Oct 3;7:12577. doi: 10.1038/s41598-017-12933-z (PMC5626680; doi:10.1038/s41598-017-12933-z)
Supplement: Supplementary file 1 — Supplementary Information [file 41598_2017_12933_MOESM1_ESM.pdf]

Discrimination of geographical origin of cultivated *Polygala tenuifolia* based on multi-element fingerprinting by inductively coupled plasma mass spectrometry

Yunsheng Zhao[1,2,3]*, Xiaofang Ma[1], Lingling Fan[1], Fuying Mao[1,2], Hongling Tian[4], Rui Xu[1], Zhe Cao[1], Xinhui Zhang[1,2], Xueyan Fu[1,2], Hong Sui[1,2,3]*

1 Ningxia Medical University Pharmacy College, Yinchuan 750004, Ningxia, China

2 Ningxia Research Center of Modern Hui Medicine Engineering and Technology, Yinchuan 750004, Ningxia, China

3 Ministry of Education Key Laboratory of Modern Hui Chinese Medicine, Yinchuan 750004, Ningxia, China

4 Institute of Industrial Crop Research, Shanxi Academy of Agricultural Sciences, Fenyang 032200, Shanxi, China

*Corresponding Author: Yunsheng Zhao (zwhjzs@126.com) and Hong Sui(13995113086@163.com)

Tel: +86-13619501878

Fax: +86-09516980193

Pharmacy College, Ningxia Medical University, No.1160 Shengli South Street, Xingqing District Yinchuan of China

Table S1. Samples source of cultivated *P. tenuifolia* in this work

Code	Locations	Coordinates	province	Code	Locations	Coordinates	province
SHX1	Suide	E110° 14′ N37° 29′	Shaanxi	SX13	Aodi	E111° 08′ N35° 26′	Shanxi
SHX2	Pucheng	E109° 35′ N34° 58′		SX14	Fenyang	E111° 49′ N37° 12′	
SHX3	Chengcheng	E109° 56′ N35° 18′		SX15	beizhang	E111° 06′ N35° 42′	
SHX4	Zizhou	E110° 03′ N37° 27′		SX16	wanan	E111° 07′ N35° 32′	
HB1	Anguo	E115° 18′ N38° 25′	Hebei	SX17	peishe	E111° 19′ N35° 17′	
HB2	Neiqiu	E114° 03′ N37° 18′		SX18	Pinglu	E111° 11′ N34° 58′	
HB3	Xinglong	E117° 31′ N40° 26′		SX19	Daning	E110° 42′ N36° 30′	
HN1	Xingyang	E113° 25′ N34° 57′	Henan	SX20	Wenxi	E111° 05′ N35° 24′	
HN2	Mengjin	E112° 25′ N34° 50′		SX21	Hongdong	E111° 38′ N36° 29′	
SX1	Pingyao	E112° 15′ N37° 10′	Shanxi	SX22	liyuan	E111° 19′ N35° 30′	
SX2	Linyi	E111° 29′ N35° 06′		SX23	dongzhen	E111° 18′ N35° 25′	
SX3	Jishan	E111° 00′ N35° 28′		SX24	Lanxian	E110° 35′ N38° 20′	
SX4	Heping	E111° 38′ N36° 28′		SX25	nianzhang	E111° 17′ N35° 15′	
SX5	Xinjiang	E111° 07′ N35° 31′		SX26	Yonghe	E110° 38′ N36° 37′	
SX6	Dongjiazhuang	E111° 45′ N32° 16′		SX27	Taiyin	E111° 35′ N35° 29′	
SX7	Shilou	E110° 47′ N37° 07′		SX28	Jiangxian	E111° 35′ N35° 36′	
SX8	tuanbo	E111° 38′ N36° 28′		SX29	Houma	E111° 18′ N35° 32′	
SX9	Xiaxian	E111° 15′ N35° 04′		SX30	hedi	E111° 21′ N35° 19′	
SX10	Xiangfen	E111° 23′ N35° 52′		SX31	Xingxian	E111° 22′ N38° 47′	
SX11	Yicheng	E111° 38′ N35° 41′		SX32	Lishi	E111° 03′ N37° 32′	
SX12	Hengqiao	E111° 13′ N35° 34′		CK	Youyu	E112° 20′ N40° 11′	

CK: *ephedra sinica* as control group.

Table S2. Elemental contents (mg/kg) in cultivated *P. tenuifolia* from different regions

	Ca	N	K	P	Mg	S	Fe	Cl	Na	Sr	Mn	Zn	B	Cu	Mo
**LOD**	0.004	0.01	0.001	0.002	0.004	0.01	0.002	0.01	0.005	0.002	0.002	0.002	0.006	0.001	0.003
**SHX1**	29567.77	20986.81	5702.76	2644.20	2057.66	1072.63	947.60	228.54	89.49	34.86	25.16	19.12	13.28	4.55	1.93
**SHX2**	32127.56	21833.30	6305.05	2064.38	1643.22	1150.38	1317.34	214.52	85.77	36.58	29.35	20.65	12.98	4.89	2.03
**SHX3**	21801.63	24359.70	7237.47	1946.25	1996.90	1070.61	617.27	232.96	97.09	33.67	29.31	19.13	14.50	4.69	1.86
**SHX4**	25672.32	21181.79	6235.94	4565.63	1873.94	992.43	710.06	187.21	90.02	30.31	20.84	15.68	17.36	4.11	1.68
**HB1**	27147.18	22192.00	6432.40	1896.88	2002.55	986.35	743.53	207.51	145.42	29.13	25.77	15.37	19.58	4.70	1.61
**HB2**	17770.21	35666.60	8379.01	2118.13	2223.01	1446.43	899.17	269.69	202.98	30.79	31.39	15.71	21.10	5.54	1.70
**HB3**	19123.79	21476.16	3381.53	1386.20	1544.26	1429.04	851.75	233.89	39.97	22.45	18.42	13.82	15.00	4.73	1.24
**HN1**	22470.69	22122.85	6211.42	1782.50	2017.34	1056.32	993.73	226.10	106.20	33.79	24.80	17.41	15.76	4.50	1.87
**HN2**	20362.39	21962.50	6362.77	1713.13	2278.13	965.11	772.10	239.06	100.10	24.30	23.05	14.50	15.85	4.36	1.35
**SX1**	24736.66	20867.55	5056.66	1948.13	1831.96	980.52	728.41	212.77	87.66	23.42	19.52	15.17	13.88	4.62	1.30
**SX2**	26037.71	28066.98	8138.69	2260.05	1682.77	1123.66	590.58	253.28	114.12	33.66	27.73	19.46	16.93	5.19	1.86
**SX3**	25705.48	29530.22	8669.09	2289.03	2351.62	1203.57	495.46	281.08	118.36	46.41	33.88	21.89	19.56	5.86	2.57
**SX4**	25848.40	23547.30	7543.55	2152.50	2324.82	1141.05	732.51	276.97	106.78	36.49	29.50	20.18	16.85	6.00	2.02
**SX5**	24363.56	24130.23	7720.40	1478.75	1645.48	1034.45	539.69	231.83	114.57	34.17	24.83	18.38	17.82	4.64	1.89
**SX6**	24623.46	20825.05	6217.71	2055.00	2369.99	1051.27	798.46	251.67	78.38	32.96	25.26	18.44	12.63	5.06	1.94
**SX7**	25213.40	19838.85	6718.52	2235.75	1699.52	954.85	659.65	199.08	76.11	32.78	27.21	17.39	13.29	4.02	1.81
**SX8**	32200.72	26119.05	7323.75	2608.13	2663.94	1205.59	909.00	234.24	100.71	45.32	28.19	21.82	15.94	4.74	2.51
**SX9**	22760.48	22230.67	5584.44	2079.75	2167.39	1057.99	794.24	191.84	98.81	27.97	24.86	17.59	14.66	4.12	1.55
**SX10**	25318.60	24649.64	7377.97	2123.25	2792.54	1133.07	1040.38	246.73	144.79	29.93	27.44	19.11	21.49	5.18	1.66
**SX11**	24718.15	23071.24	6999.13	2811.25	1690.22	1197.51	926.42	203.98	89.10	30.45	29.49	20.43	13.22	4.46	1.69
**SX12**	25505.58	21077.67	6219.49	2632.50	2296.50	1073.89	1053.83	197.99	91.96	28.00	25.72	18.01	13.65	4.08	1.55
**SX13**	26685.94	20668.50	6270.21	2463.13	1887.70	1097.13	1051.60	206.97	88.46	28.61	25.99	17.99	13.13	4.21	1.58
**SX14**	25896.18	22374.39	5716.25	2089.88	1804.73	909.05	1096.41	179.57	102.48	27.37	23.80	15.66	15.21	3.66	1.52
**SX15**	24880.38	22665.39	7362.32	2423.25	1708.60	978.18	859.49	194.46	108.00	28.92	24.92	17.81	16.03	3.95	1.60
**SX16**	22289.54	20652.53	6565.37	2043.75	1543.25	1131.12	922.64	237.21	79.25	28.18	24.17	17.01	14.76	4.32	1.56
**SX17**	28884.64	27659.58	8191.10	2847.50	1984.18	1097.19	1120.98	240.08	112.70	38.14	29.31	20.89	16.72	5.09	2.11
**SX18**	24888.04	23148.81	7122.67	2346.88	2461.85	966.44	1078.32	266.13	115.10	29.14	25.04	17.64	16.08	5.34	1.61
**SX19**	27726.64	21380.02	5822.35	1940.00	1955.66	1010.86	1112.36	228.20	80.91	28.52	23.41	17.78	12.90	4.17	1.58
**SX20**	26689.86	23358.41	7186.25	1885.00	1774.03	1123.01	916.69	230.62	93.62	35.93	26.77	19.12	13.89	4.91	1.99
**SX21**	26110.68	20506.82	5346.31	2865.63	1949.38	1039.07	1200.87	211.15	68.28	32.82	22.58	17.73	14.13	4.19	1.82
**SX22**	28528.98	21572.64	6544.50	1950.83	1832.05	1104.74	1015.76	199.81	86.53	33.45	25.85	18.12	12.84	4.16	1.85
**SX23**	25870.53	22275.27	6793.77	1897.98	2020.92	996.28	839.53	206.27	101.20	31.22	23.82	17.22	15.02	3.67	1.73
**SX24**	20680.40	19998.67	5223.67	1912.70	1897.10	939.43	671.98	188.09	79.10	22.84	20.13	14.86	13.74	3.29	1.26
**SX25**	29097.71	21667.04	6173.14	1925.00	1724.63	1100.34	911.66	194.84	113.41	34.84	25.74	18.95	16.83	3.87	1.93
**SX26**	2579.70	29580.55	8373.86	2025.00	1471.93	1031.83	998.69	226.65	116.31	32.23	26.69	17.99	17.90	4.72	1.78
**SX27**	29381.06	20405.26	6121.35	1963.65	1982.99	1092.54	888.20	239.02	72.03	30.26	25.71	18.74	11.79	4.36	1.68
**SX28**	25317.72	19804.07	6299.41	2456.13	2354.44	943.77	825.07	207.89	101.67	24.31	27.91	14.76	15.99	4.21	1.35
**SX29**	26906.65	23033.17	6897.58	1979.10	1616.74	1116.00	923.68	243.90	96.33	33.85	27.99	20.29	17.29	4.81	1.87
**SX30**	27364.07	20762.27	6928.66	2233.88	1745.34	975.85	1153.56	232.20	85.45	27.98	33.29	16.68	18.68	4.59	1.55
**SX31**	22187.25	21201.78	5480.93	1862.43	1771.94	880.07	1148.75	204.65	117.80	23.09	29.18	13.77	17.70	4.26	1.28
**SX32**	23454.47	26505.37	6297.05	2378.44	1635.11	919.34	856.48	258.15	97.80	25.33	24.84	15.96	15.51	5.25	1.40
**RSD(%)**	12.32	13.44	15.70	23.02	16.59	11.47	21.22	11.56	25.46	17.10	12.77	11.86	14.87	12.74	17.16
**CK**	12674.39	1798.69	1294.9	916.43	2369.99	1370.41	100.57	73.84	85.02	10.79	11.01	3.83	4.84	1.12	0.62

CK：*ephedra sinica* as control group.

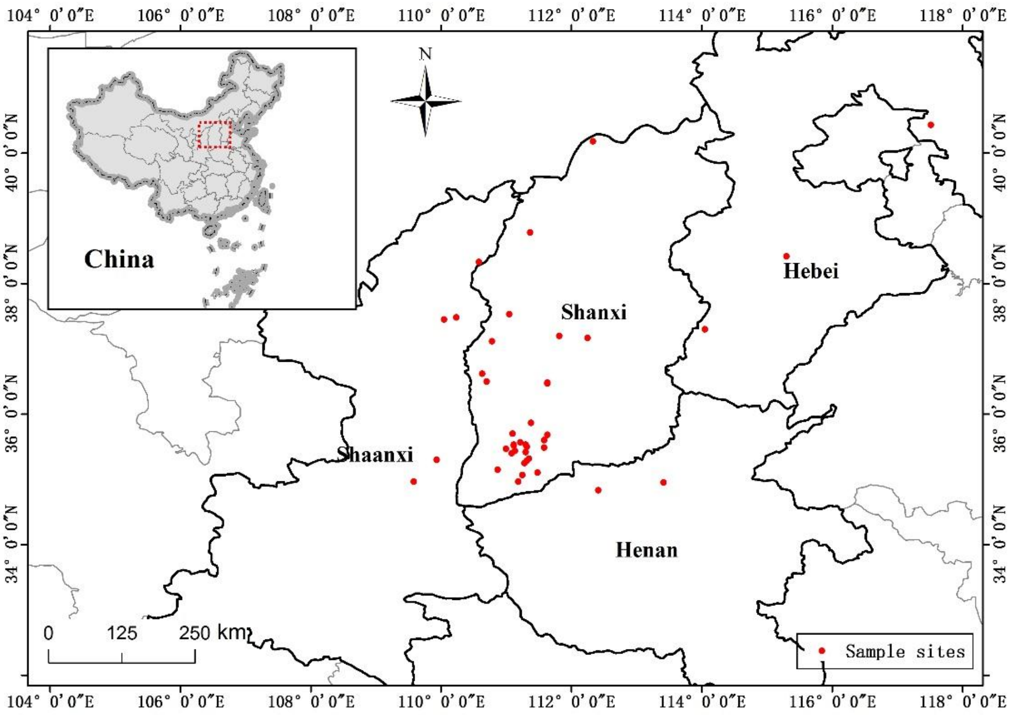

Fig. S1. Geographical origin locations of the 41 cultivated *P. tenuifolia* samples involved in four provinces for this study. Maps generated using ArcGIS 10.3.
(ArcMap URL: http://www.esri.com/en/arcgis/products/arcgis-pro/resources/arcmap-resources)

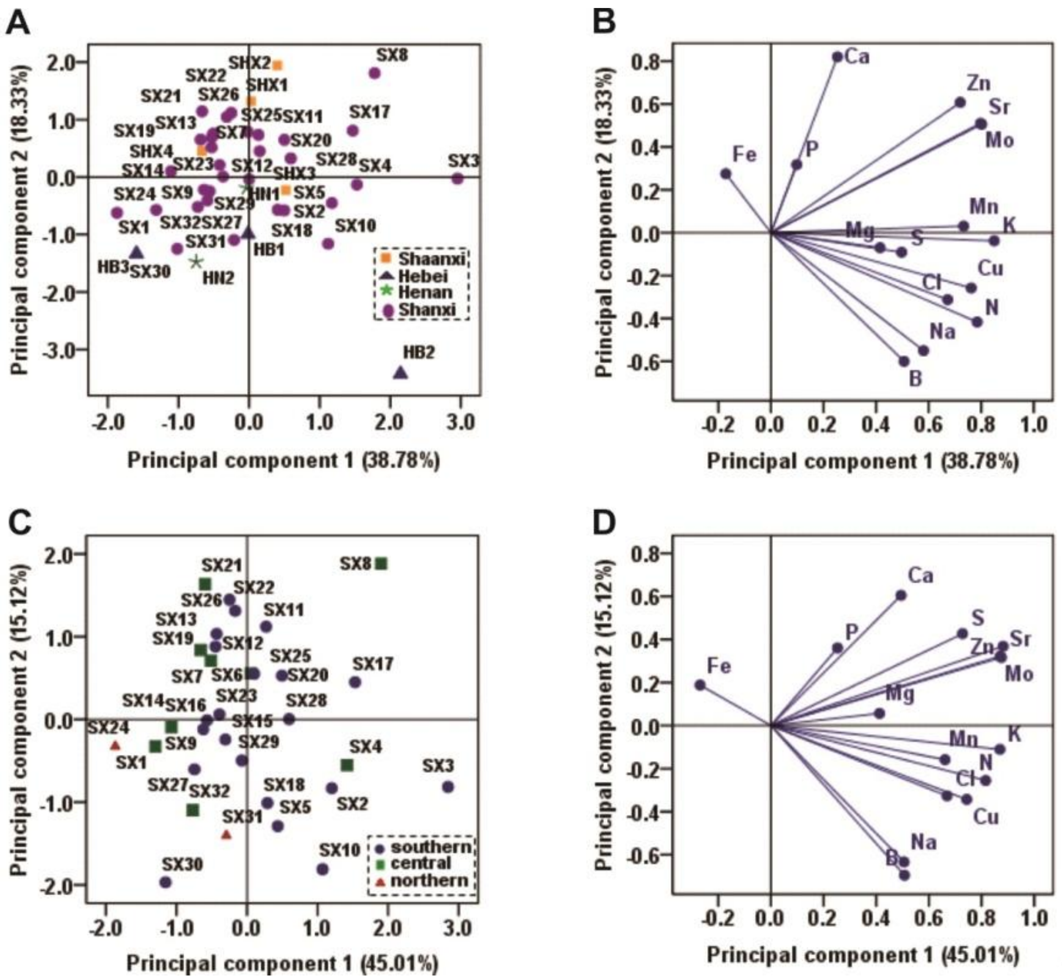

Fig. S2. Principal component analysis on elemental concentrations based on 15 variables in cultivated *P. tenuifolia*. Panel a and b illustrate the score plot and the corresponding loading plot of 41 cultivated *P. tenuifolia* samples from different geographical origins. Grouping according to geographical location is shown by principle components 1 and 2, which explained 38.78% and 18.33% of the variance. Panel c and d illustrate the score plot and the corresponding loading plot of 32 cultivated *P. tenuifolia* samples from different regions in Shanxi. Grouping according to geographical location is shown by principle components 1 and 2, which explained 45.01% and 15.12% of the variance.
